# Usefulness of the triglyceride glucose-body mass index in evaluating nonalcoholic fatty liver disease: insights from a general population

**DOI:** 10.1186/s12944-021-01506-9

**Published:** 2021-07-28

**Authors:** Rongsheng Wang, Longlong DAI, Yanjia Zhong, Guobo Xie

**Affiliations:** 1 Department of Intensive Care Unit, Jiangxi Provincial People’s Hospital Affiliated to Nanchang University, Nanchang, P.R. China 330006; 2 Department of Cardiology, Ruichang People’s Hospital, Jiujiang, P.R. China 332200; 3 Department of Endocrinology, Jiangxi Provincial People’s Hospital Affiliated to Nanchang University, Nanchang, P.R. China 330006; 4 Department of Cardiology, Jiangxi Provincial People’s Hospital Affiliated to Nanchang University, Nanchang, P.R. China 330006

**Keywords:** Triglyceride, Body mass index, Triglyceride, Triglyceride glucose-body mass index, Triglyceride-glucose index

## Abstract

**Background:**

Triglyceride glucose-body mass index (TyG-BMI) is a recently developed alternative indicator to identify insulin resistance. However, few studies have investigated the association between the TyG-BMI and nonalcoholic fatty liver disease (NAFLD). Therefore, this study aimed to study the relationship between NAFLD and the TyG-BMI in the general population and its predictive value.

**Methods:**

A cross-sectional study was conducted on 14,251 general subjects who took part in a comprehensive health examination. The anthropological characteristics and many risk factors for NAFLD were measured.

**Results:**

After fully adjusting for confounding variables, a stable positive correlation was found between NAFLD and the TyG-BMI (OR: 3.90 per SD increase; 95% CI: 3.54 to 4.29; *P*-trend< 0.00001). This positive correlation was not simply linear but a stable non-linear correlation. Additionally, obvious threshold effects and saturation effects were found, in which a threshold effect occurred when the TyG-BMI was between 100 and 150; when the TyG-BMI was between 300 and 400, the corresponding NAFLD risk appeared saturated. Furthermore, receiver operating characteristic analysis showed that the TyG-BMI could better predict the risk of NAFLD than other traditional indicators [TyG-BMI (AUC): 0.886; 95% CI: 0.8797–0.8927; *P* < 0.0001], particularly among young and middle-aged and non-obese people.

**Conclusions:**

This epidemiological study is the first on the association between the TyG-BMI and NAFLD risk in the general population. In this large data set from the general population, the TyG-BMI showed an independent positive correlation with NAFLD. The discovery of the threshold effect and saturation effect between them provides a new idea to prevent and treat NAFLD.

**Supplementary Information:**

The online version contains supplementary material available at 10.1186/s12944-021-01506-9.

## Background

Nonalcoholic fatty liver disease (NAFLD) is the most common and fastest-growing liver disease. Recent epidemiological surveys have estimated the global prevalence of NAFLD at approximately 25%, which is expected to increase to 33.5% by 2030 [1–3]. NAFLD may develop into nonalcoholic steatohepatitis, compensated/decompensated cirrhosis, or even hepatocellular carcinoma [1, 4], while extrahepatic NAFLD can aggravate cardiovascular and cerebrovascular diseases, diabetes, and kidney disease with adverse consequences [5–7]. Despite the increasing prevalence of NAFLD and its adverse effects on multiple systems throughout the body, no definite drug treatment currently exists except for lifestyle changes through exercise [8]. Therefore, identifying the population prone to NAFLD early through simple and effective diagnostic tools in daily practice is beneficial.

Insulin resistance (IR) refers to reduced peripheral tissue insulin sensitivity, characterized by impaired glucose uptake and oxidation, and it participates in the key link of the pathogenesis of NAFLD [8, 9]. The triglyceride-glucose (TyG) index is a compound index that combines fasting blood glucose (FPG) and fasting triglyceride (TG) and can better reflect IR. Because of its convenient acquisition and simple calculation, it has been widely accepted and used clinically [10–12]. In a recent study, Er et al. found that the combination of the body mass index (BMI) and TyG index can simultaneously reflect the information of many important clinical indexes, such as blood glucose, blood lipids and the BMI, and can better reflect IR than the TyG index alone [13]. Considering the importance of IR in the pathogenesis of NAFLD [8, 9], researchers have speculated that the TyG-BMI may be a good marker to predict NAFLD in the general population. The existing several studies of separately considering the non-obese or overweight, obese people [14–16]. However, it remains unclear in which BMI population the TyG-BMI has better NAFLD identification ability, and whether the TyG-BMI and NAFLD are linear or non-linear associations is also an unresolved clinical issue. Therefore, this study aimed to study the association between the TyG-BMI and NAFLD in the general population and its predictive value, analyse the relationship between them in detail and the interaction with other factors and provide new ideas to prevent and treat NAFLD.

## Methods

### Study design and subjects

The NAGALA study is an ongoing cohort study, and the design details of the study have been described in previous studies [17]. Briefly, the NAGALA study project recruited 20,944 general subjects who participated in a comprehensive health examination at Murakami Memorial Hospital from 2004 to 2015. The project aimed to assess risk factors for common chronic diseases from health check-up information in the general population.

This study was a post hoc analysis of the public data in the NAGALA cohort, and the original data were uploaded to the DRYAD database by Professor Fukui [18]. To make the data better serve the human population, according to the content of the public data usage protocol of the Dryad database, each researcher can use the public data of the database for secondary analysis.

Based on previous studies, this study established a new hypothesis: in the general population, is TyG-BMI and NAFLD related? According to the purpose of the study, a cross-sectional design was adopted, and the following exclusion criteria were established: 1) subjects diagnosed with viral hepatitis or impaired fasting glucose or diabetes at baseline (*N* = 1547); 2) subjects who were taking medication at baseline (*N* = 2321); 3) subjects with incomplete covariable data (*N* = 873); 4) alcohol consumption: male ≥210 g/w or female ≥140 g/w [19]. Because Murakami Memorial Hospital’s institutional ethics committee had authorized the previous study, separate ethical approval was no longer required for this study [17]. Additionally, because the identification information of the subjects was deleted from the data and uniformly replaced by the health check code and the informed consent for each subject on using the data in the previous study was obtained, the application to obtain informed consent again was not required in this study. The entire study protocol followed the Declaration of Helsinki.

### Health check-up and laboratory measurement

The basic health check-up information of the subjects was recorded by trained medical staff using a standard and unified questionnaire, including height, sex, habit of exercise, systolic/diastolic blood pressure (S/BBP), age, smoking status, waist circumference (WC), weight, and drinking status. Blood samples for biochemical analysis were obtained after an overnight fasting period of at least 8 h. Analysis indicators included alanine aminotransferase (ALT), haemoglobin A1c (HbA1c), TG, gamma-glutamyl transferase (GGT), total cholesterol (TC), aspartate aminotransferase (AST), high-density lipoprotein cholesterol (HDL-C) and FPG.

### Definitions and calculations

BMI = weight/height^2^. TyG = Ln [(FPG (mg/dL)/2) × TG (mg/dL) ×] [11]. TyG-BMI = BMI × TyG [13]; drinking status: according to the amount of alcohol consumed per week, classified as non or small (< 40 g/w), light (40–139 g/w) and moderate (140–209 g/w); smoking status: at baseline data access, classified as nons/former/current smokers based on smoking history; Habit of exercise: Defined as at least once a week to attend a physical exercise.

### Diagnosis of NAFLD by abdominal ultrasonography

After abdominal ultrasonography was conducted by a trained technician, experienced gastroenterologists examined the sonograms and made a comprehensive judgement and NAFLD diagnosis based on the sonographic features of the four types of deep attenuation, ultrasonic hepatic vascular imaging, ultrasound comparison of the liver and kidney and brightness of the liver under ultrasound without knowing the information of the participants [20].

### Statistical analysis

This study makes a statistical analysis of TyG-BMI as a categorical variable (the quintile of the TyG-BMI was calculated using a quantile function divided into five groups) and a continuous variable to deeply understand the association between NAFLD and the TyG-BMI. The main statistical analysis process was as follows:

Step one (statistical description and analysis of the baseline data): Continuous variables in this study were described as the mean [standard deviation (SD)] or median (interquartile range), and the differences among TyG-BMI groups were evaluated by the Kruskal Wallis H test and Steel Dwass test or one-way ANOVA and Tukey’s HSD test. Categorical variables were described as frequencies (%), and chi-squared test was used to check the differences between the TyG-BMI groups.

Step two (association analysis of TyG-BMI and NAFLD): Before establishing the multivariable logical regression model, the researchers first checked the collinearity between the covariables (Supplementary Table 1) [21]. Next, the correlation between the baseline data and TyG-BMI was checked by linear regression (Supplementary Table 2) [22]. The variables significantly related to TyG-BMI were considered auxiliary factors of the relationship between the two. These auxiliary factors will be included as important adjustment variables in the multivariate regression model. After the above steps were completed, multiple logistic regression was used to calculate the odds ratio (OR) and 95% confidence interval (CI) before and after adjustment to further explore the association between NAFLD and TyG-BMI. To further ensure the stability of the data analysis, the researchers further processed the TyG-BMI quintile as a continuous variable to conduct a trend test. In this study, adjustment of the multivariable logical regression model follows the STROBE statement [23] and shows the results according to different adjustment methods and adjustment degrees through several models, in which the crude model does not adjust any covariates; model 1 adjusted for general demographic variables; model 2 adjusted the non-collinear variables in which the impact of TyG-BMI on NAFLD risk was more than 10% and the non-collinear variable of *P* < 0.05 in univariate analysis [24]; model 3 adjusted for non-collinear variables associated with TyG-BMI. Additionally, the research team used the generalized additive model to analyse the smoothing function to simulate the potential non-linear relationship between NAFLD and the TyG-BMI.

Step three (Hierarchical analysis): To verify whether the correlation between the TyG-BMI and NAFLD was different among different populations, the research team conducted exploratory hierarchical analysis using a hierarchical logistic regression model in some subgroups and examined the differences between different stratifications using a likelihood ratio test to determine whether an interaction occurred.

Step four (ROC curve analysis of NAFLD-related indicators): Previous research has shown that the BMI, WC, lipids, the lipid ratio, blood glucose, the TyG index and related parameters of NAFLD have better prediction performance [16, 25, 26]. In this study, the predictive value of WC, the BMI, the TyG-WC, TyG index, TG/HDL-C ratio, the TyG-BMI and other factors related to NAFLD was analyzed by constructing receiver operating characteristic curve (ROC).

All the statistical analysis processes were based on the R language-based Empower (version 2.0) statistical software, with a 2-sided significance threshold of *p* < 0.05.

## Results

### Basic clinical and laboratory characteristics

In the present study, 14,251 eligible subjects were evaluated. According to the quintile of the TyG-BMI, the subjects were equally divided into 5 groups. The clinical and laboratory characteristics of each TyG-BMI group are recorded in Table 1. The average age of the subjects was 43.53 years, 52% of the subjects were male, and the prevalence rate of NAFLD was 17.59%. The subjects with a higher TyG-BMI value had a higher male proportion, an older age, a higher BMI, a higher WC, a higher TyG index, a higher blood pressure, a higher prevalence of NAFLD, a higher proportion of smoking and drinking, and fewer people who maintained exercise habits. Regarding the laboratory parameters, except for HDL-C and TyG-BMI that showed an inverse trend, most of the other laboratory parameters also maintained a trend similar to that of the clinical indicators; among the participants with higher TyG-BMI values, AST, HbA1c, TC, GGT, FPG, ALT, and TG were higher.

**Table 1:** Baseline characteristics of five groups

	TyG-BMI quintile					
	Q1(97.48–147.69)	Q2(147.72–164.94)	Q3(164.95–182.05)	Q4(182.07–205.33)	Q5(205.34–421.32)	*P*-value
No. of participants	2850	2850	2851	2849	2851	
Age (years)	40.18 (8.39)	42.98 (8.70)	44.39 (8.93)^*^	45.35 (8.97)^*^	44.77 (8.50)	< 0.001
Weight (kg)	48.32 (5.59)	54.08 (6.16)	59.33 (6.80)	65.00 (7.04)	74.57 (10.04)	< 0.001
Height (cm)	161.50 (7.36)	163.06 (8.31)	164.94 (8.62)	166.74 (8.33)	167.74 (8.16)	< 0.001
BMI (kg/m^2^)	18.49 (1.30)	20.29 (1.11)	21.75 (1.14)	23.34 (1.26)	26.45 (2.56)	< 0.001
WC (cm)	66.42 (4.84)	71.14 (4.99)	75.57 (5.11)	80.26 (5.01)	87.54 (6.92)	< 0.001
Sex (male)	538 (18.88%)	1046 (36.70%)	1548 (54.30%)	2034 (71.39%)	2245 (78.74%)	< 0.001
NAFLD	11 (0.39%)	56 (1.96%)	198 (6.94%)	612 (21.48%)	1630 (57.17%)	< 0.001
Habit of exercise	438 (15.37%)	548 (19.23%)	547 (19.19%)	497 (17.44%)	440 (15.43%)	< 0.001
ALT (IU/L)	13 (11–17)	14 (11–18)	16 (12–20)	19 (15–25)	25 (18–35)	< 0.001
AST (IU/L)	16 (13–19)^**^	16 (13–19)	16 (14–20)	18 (14–21)	20.00 (16–25)	< 0.001
GGT (IU/L)	12 (10–14)	13 (10–16)	14.00 (11–19)	17.00 (13–24)	22.00 (16–33)	< 0.001
HDL-C (mmol/L)	1.73 (0.39)	1.62 (0.38)	1.48 (0.36)	1.32 (0.31)	1.15 (0.27)	< 0.001
TC (mmol/L)	4.73 (0.78)	4.99 (0.81)	5.11 (0.86)	5.28 (0.82)	5.50 (0.88)	< 0.001
TG (mmol/L)	0.41 (0.30–0.53)	0.56 (0.44–0.73)	0.71 (0.55–0.93)	0.95 (0.72–1.24)	1.42 (1.03–1.94)	< 0.001
FPG (mmol/L)	4.88 (0.37)	5.03 (0.38)	5.16 (0.38)	5.27 (0.36)	5.40 (0.36)	< 0.001
TyG	7.33 (0.45)	7.73 (0.39)	7.98 (0.40)	8.29 (0.41)	8.72 (0.49)	< 0.001
HbA1c (%)	5.10 (4.90–5.35)	5.10 (4.91–5.40)^***^	5.10 (4.90–5.40)	5.20 (5.00–5.40)	5.20 (5.00–5.50)	< 0.001
SBP (mmHg)	104.98 (12.14)	108.99 (12.62)	113.40 (12.96)	117.90 (13.62)	124.40 (14.44)	< 0.001
DBP (mmHg)	64.91 (8.30)	67.66 (9.06)	70.55 (9.22)	74.01 (9.56)	78.48 (9.93)	< 0.001
Drinking status						< 0.001
Non or small	2606 (91.44%)	2451 (86.00%)	2305 (80.85%)	2197 (77.11%)	2246 (78.78%)	
Light	209 (7.33%)	307 (10.77%)	406 (14.24%)	434 (15.23%)	402 (14.10%)	
Moderate	35 (1.23%)	92 (3.23%)	140 (4.91%)	218 (7.65%)	203 (7.12%)	
Smoking status						< 0.001
Non	2299 (80.67%)	2018 (70.81%)	1732 (60.75%)	1387 (48.68%)	1310 (45.95%)	
Former	246 (8.63%)	406 (14.25%)	529 (18.55%)	690 (24.22%)	688 (24.13%)	
Current	305 (10.70%)	426 (14.95%)	590 (20.69%)	772 (27.10%)	853 (29.92%)	

Values were expressed as mean (SD) or medians (quartile interval) or n (%). Abbreviations: NAFLD: Nonalcoholic fatty liver disease; *BMI* body mass index; *TyG* the triglyceride-glucose index; *TyG-BMI* triglyceride glucose-body mass index; *WC* Waist circumference; *ALT* alanine aminotransferase; *AST* aspartate aminotransferase; *GGT* gamma-glutamyl transferase; *HDL-C* high-density lipoprotein cholesterol; *TC* total cholesterol; *TG* triglyceride; *HbA1c* hemoglobin A1c; *FPG* fasting plasma glucose; *SBP* systolic blood pressure; *DBP* Diastolic blood pressure. *P > 0.05 vs Q5. **P > 0.05 vs Q2. ***P > 0.05 vs Q1, Q3. Other variables with no special mark on the upper right corner had *P* values < 0.05 after pair-wise comparison

### Association analysis of NAFLD and the TyG-BMI

As described in the second step of statistical analysis, the association between NAFLD and the TyG-BMI was mainly analysed in multiple models using the TyG-BMI as a categorical variable and a continuous variable. In the crude model, NAFLD and TyG-BMI was positively related to relationship in the general population; and with the gradual increase of TyG-BMI, the NAFLD risk was gradually increased (Table 2). In multivariate analysis, after adjusting the non-collinear general demographic data in model 1, the positive correlation and trend between the TyG-BMI and NAFLD remained unchanged (OR: 5.40 per SD increase; 95% CI: 5.01 to 5.83; *P*-trend< 0.00001). Model 2 adjusted the non-collinear variables in which the impact of the TyG-BMI on NAFLD risk was more than 10% and the non-collinear variables in univariate analysis with *P* value less than 0.05. The OR values of the TyG-BMI and NAFLD were slightly lower than those in model 1, and the positive correlation direction and trend of the core results were maintained (OR: 3.87 per SD increase; 95% CI: 3.51 to 4.26; *P*-trend< 0.00001). In model 3, after adjusting the non-collinear variables related to the TyG-BMI, it was found that the NAFLD risk increased by 290% for per 1 SD increase in TyG-BMI (95% CI: 3.54 to 4.29; *P*-trend< 0.00001). In four different models, the core direction of the relationship between NAFLD and the TyG-BMI did not change significantly, and the results of sensitivity analysis further verified the stability of the positive correlation between them.

**Table 2:** Logistic regression analyses for the association between TyG-BMI and incident NAFLD in different models

	Odds ratios (95% confidence interval)			
	Crude model	Model 1	Model 2	Model 3
TyG-BMI(per SD increase)	6.17 (5.75, 6.62)	5.40 (5.01, 5.83)	3.87 (3.51, 4.26)	3.90 (3.54, 4.29)
TyG-BMI (Quintile)				
Quintile 1	Ref	Ref	Ref	Ref
Quintile 2	5.17 (2.70, 9.89)	4.25 (2.22, 8.15)	4.04 (2.03, 8.03)	4.03 (2.03, 8.01)
Quintile 3	19.26 (10.47, 35.43)	13.33 (7.22, 24.60)	10.72 (5.58, 20.62)	10.81 (5.63, 20.75)
Quintile 4	70.61 (38.80,128.51)	42.25 (23.09, 77.30)	25.09 (13.11,47.99)	25.52 (13.36, 48.74)
Quintile 5	344.54 (189.71,625.75)	186.82 (102.20,341.51)	72.84 (37.82,140.30)	74.76 (38.86,143.79)
*P*-trend	< 0.0001	< 0.0001	< 0.0001	< 0.0001

Abbreviations: *TyG-BMI*: triglyceride glucose-body mass index;

Model 1 adjusted for sex, age, habits of exercise, drinking status. Smoking status, SBP, and height;

Model 2 adjusted for sex, age, ALT, AST, habits of exercise, GGT; HDL-C, TC, TG, FPG, HbA1c, smoking status, SBP and height;

Model 3 adjusted for sex, age, ALT, AST, habits of exercise, GGT; HDL-C, TC, TG, FPG, HbA1c, smoking status, drinking status, SBP and height

### Non-linear relationship analysis between NAFLD and the TyG-BMI

**Fig. 1:**
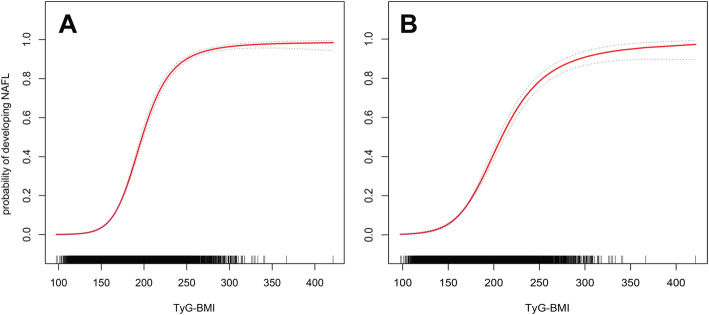
Non-linear relationship between the TyG-BMI and NAFLD in the unadjusted model (**A**) and adjusted model (**B**). Dashed lines indicate 95% confidence intervals. Adjusted for sex, age, ALT, AST, habits of exercise, GGT, HDL-C, TC, TG, FPG, HbA1c, smoking status, SBP and height

To further explore TyG-BMI and non-linear relation between NAFLD, the team performed smoothing function analysis using a generalized additive model to simulate the potential non-linear association between NAFLD and the TyG-BMI (Fig. 1). Before and after adjustment, the model maintained the stability of the non-linear association between the TyG-BMI and NAFLD, and threshold and saturation effects were found in the correlation between NAFLD and the TyG-BMI. A TyG-BMI between 100 and 150 likely indicates a threshold effect point, and a TyG-BMI between 300 and 400 suggests the corresponding NAFLD risk may indicate a saturation effect.

### Subgroup analysis

In subgroup analysis (Table 3), a significant interaction was found between age, the BMI and the TyG-BMI-related NAFLD risk (*P*-interaction< 0.05), while the interaction between sex, the smoking status, habit of exercise, the drinking status and the TyG-BMI indicated no significant significance. Age stratification analysis showed that middle-aged people had a higher risk of TyG-BMI-related NAFLD than other age groups; BMI stratification also found an interesting result: among non-obese people, the risk of NAFLD related to the TyG-BMI was higher than that of overweight and obese people.

**Table 3:** Stratified associations between TyG-BMI and NAFLD by age, sex, habit of exercise, BMI, smoking status and drinking status

Subgroup	No. of participants	unadjusted OR (95% CI)	adjusted OR (95% CI)	*P-*interaction
Age (years)				0.0095
18–30	645	6.12 (4.04, 9.27)	3.68 (2.35, 5.76)	
31–45	8210	6.98 (6.33, 7.70)	4.31 (3.82, 4.87)	
46–60	4909	5.14 (4.60, 5.74)	3.30 (2.90, 3.76)	
> 60	487	5.35 (3.59, 7.98)	3.86 (2.53, 5.89)	
Sex				0.1101
Male	7411	5.50 (5.04, 6.00)	3.68 (3.28, 4.12)	
Female	6840	5.96 (5.25, 6.76)	4.20 (3.64, 4.83)	
Habit of exercise				0.0667
Yes	2470	6.88 (5.71, 8.30)	4.58 (3.72, 5.64)	
No	11,781	6.04 (5.60, 6.52)	3.76 (3.41, 4.16)	
BMI (kg/m^2^)				0.0002
< 24	10,881	10.02 (8.65, 11.59)	5.82 (4.75, 7.14)	
≥ 24, < 28	2726	4.58 (3.86, 5.44)	2.81 (2.21, 3.56)	
≥ 28	644	3.97 (2.79, 5.66)	2.89 (1.97, 4.25)	
Smoking status				0.5652
Non	8746	6.59 (5.98, 7.27)	4.01 (3.56, 4.51)	
Former	2559	5.78 (4.95, 6.75)	3.74 (3.13, 4.47)	
Current	2946	5.46 (4.75, 6.29)	3.66 (3.12, 4.30)	
Drinking status				0.5258
Non or small	11,805	6.43 (5.95, 6.96)	3.95 (3.57, 4.37)	
Light	1758	5.45 (4.47, 6.64)	3.47 (2.78, 4.33)	
Moderate	688	5.88 (4.21, 8.21)	4.05 (2.83, 5.80)	

Abbreviations: *OR* Odds ratios; other abbreviations as in Table 1

Adjusted for sex, age, ALT, AST, habits of exercise, GGT; HDL-C, TC, TG, FPG, HbA1c, smoking status, SBP and height

### ROC analysis

**Fig. 2:**
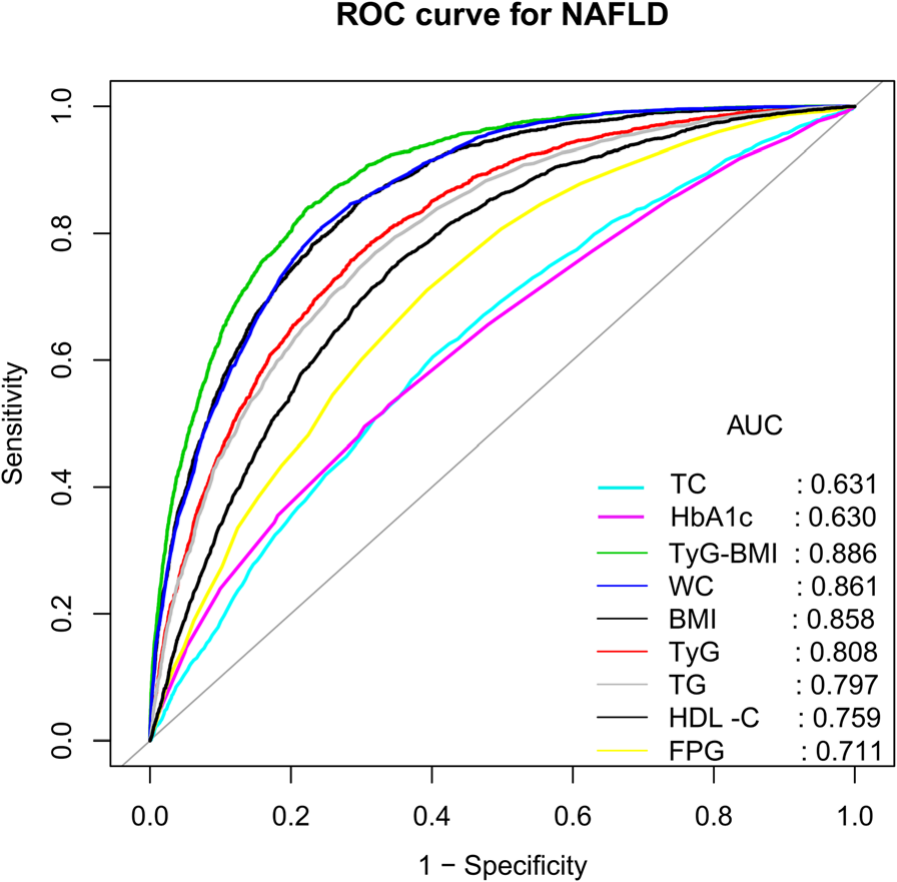
ROC curve analysis of NAFLD-related indicators. Receiver operating characteristic (ROC), NAFLD: nonalcoholic fatty liver disease; BMI: body mass index; TyG: triglyceride-glucose index; TyG-BMI: triglyceride glucose-body mass index; WC: waist circumference; HDL-C: high-density lipoprotein cholesterol; TC: total cholesterol; TG: triglyceride; HbA1c: haemoglobin A1c; FPG: fasting plasma glucose

Figure 2 shows the ROC curve of the ability of the TyG-BMI and traditional indicators to predict NAFLD risk. Table 4 shows the results of the area under the curve (AUC) in ROC analysis, in which the AUC of the TyG-BMI was 0.886 (95% CI: 0.8797–0.8927), significantly higher than that of the single TyG index, TG/HDL ratio, the BMI, and traditional markers such as TG, FPG, WC, HDL-C, HbA1c, and TC (*P* < 0.0001). Furthermore, TyG-WC showed that the area under the ROC curve to detect NAFLD was second only to the TyG-BMI, and no significant difference was found between them (*P* = 0.3662). Considering that significant differences were found in age and BMI stratification in subgroup analysis, the predictive performance of the TyG-BMI was further evaluated in different age stratifications and BMI stratifications for NAFLD by ROC analysis, revealing that the TyG-BMI had a larger AUC to predict NAFLD in young and middle-aged and non-obese people (Table 5). Additionally, studies on TyG-WC also used the same analysis steps, demonstrating that TyG-WC also had a high predictive value for NAFLD in non-obese people and young and middle-aged people.

**Table 4:** Areas under the receiver operating characteristic curves for each evaluated parameters in identifying nonalcoholic fatty liver disease

	AUC	95% confidence interval	Best threshold	Specificity	Sensitivity
BMI	0.858^*^	0.8503–0.8651	22.5521	0.7015	0.8524
TyG	0.808^*^	0.7996–0.8173	8.2059	0.7149	0.7575
TyG-BMI	0.886	0.8797–0.8927	189.6932	0.7787	0.8381
WC	0.861^*^	0.8539–0.8681	79.6500	0.7571	0.8085
TyG-WC	0.885	0.8782–0.8911	637.8369	0.7207	0.8827
TC	0.631^*^	0.6191–0.6427	5.2108	0.5985	0.6051
HbA1c	0.630^*^	0.6175–0.6419	5.3250	0.6956	0.4950
FPG	0.711^*^	0.7005–0.7217	5.1902	0.6073	0.7100
TG	0.797^*^	0.7877–0.8061	0.8411	0.6799	0.7698
HDL-C	0.759^*^	0.7490–0.7686	1.3408	0.6478	0.7547
TG/HDL-C	0.815^*^	0.8060–0.8233	0.6119	0.6818	0.8046

Abbreviations: *AUC* area under the curve; other abbreviations as in Table 1. *P < 0.0001, compare with TyG-BMI

**Table 5:** Results of the receiver operating characteristic curve analyses of TyG-BMI and TyG-WC for predicting NAFLD in subgroups of age and BMI

	AUC	95%CI low	95%CI upp	Best threshold	Specificity	Sensitivity
**TyG-BMI**						
Age (years)						
> 60	0.8247	0.7778	0.8715	196.7369	0.7956	0.7531
46–60	0.8440	0.8311	0.8569	196.4398	0.7978	0.7271
31–45	0.9090	0.9015	0.9164	189.6580	0.8057	0.8714
18–30	0.9258	0.8908	0.9608	172.4438	0.7599	0.9268
BMI (kg/m^2^)						
≥ 28	0.7150	0.6696	0.7604	254.8981	0.7296	0.6000
≥ 24, < 28	0.7070	0.6876	0.7264	216.5987	0.7008	0.5998
< 24	0.8457	0.8335	0.8579	178.0661	0.7658	0.7776
**TyG-WC**						
Age (years)						
> 60	0.7942	0.7439	0.8445	685.9048	0.7537	0.7037
46–60	0.8426	0.8298	0.8554	666.8453	0.7436	0.7818
31–45	0.9080	0.9006	0.9154	625.1197	0.7995	0.8621
18–30	0.9393	0.9110	0.9676	619.1378	0.8493	0.9024
BMI (kg/m^2^)						
≥ 28	0.7346	0.6891	0.7800	806.3716	0.7547	0.6186
≥ 24, < 28	0.7099	0.6906	0.7292	711.0072	0.6313	0.6988
< 24	0.8528	0.8413	0.8643	620.9366	0.7500	0.8153

Abbreviations: *AUC* area under the curve; other abbreviations as in Table 1

## Discussion

NAFLD not only causes a series of pathological changes of the liver, but also leads to the occurrence and development of a variety of extrahepatic diseases, which has become an important risk factor for a variety of metabolism-related diseases [2, 5–7]. This study reported for the first time a stable and independent positive association between NAFLD and the TyG-BMI in the general population that is not simply linear but non-linear and has threshold and saturation effects.

### Comparison with other studies

IR is the key factor in the pathogenesis of NAFLD [8, 9], in which blood lipid parameters, the blood lipid ratio, blood glucose parameters, the BMI and WC were often used to evaluate the IR status. Recently, an increasing number of studies have found that the combination of FPG and TG (TyG) can better identify IR, likely related to the TyG index considering both blood glucose and blood lipids, and the TyG index reflecting the metabolic status of the body more comprehensively [10–12]. The BMI is the most important anthropometric index commonly used to evaluate obesity clinically. In 2016, Er et al. found that the new index formed by combining the TyG index with the BMI (TyG-BMI) can better reflect the state of IR. Compared with lipid parameters, the lipid ratio, blood glucose parameters, the TyG index and obesity-related parameters, the TyG-BMI has the largest AUC in identifying IR [13]. In several follow-up studies, the Tuo and Lim teams have confirmed this finding [27, 28]. Additionally, recently, the TyG-BMI has been found to be strongly associated with cardiovascular and cerebrovascular diseases such as hypertension and ischaemic stroke [29, 30]. On the other hand, the TyG-BMI also has a similar positive correlation with NAFLD and shows a better predictive performance than other traditional indicators [14–16]. In a cross-sectional survey of 6809 healthy non-obese people, Zhang et al. first found that TyG-BMI is positively correlated with NAFLD and can better identify non-obese NAFLD than the TyG index, BMI, TG and FPG [14]. This finding suggests that the TyG-BMI may be a useful marker to predict NAFLD and may provide benefits to prevent and treat NAFLD. Follow-up studies by Li et al. also confirmed this finding that the TyG-BMI can be used to identify the NAFLD risk in non-obese people and may be at a higher risk in women [15]. In addition, a recent study by Khamseh et al. found that the TyG-WC and TyG-BMI are associated with NAFLD in overweight and obese people, and the correlation between liver fibrosis and TyG-BMI was stronger [16]. Compared with these similar studies, this study considered non-obese individuals, overweight individuals and obese individuals in the general population and continued to expand the sample size based on previous studies. The results of this study not only verified the correlation between NAFLD and the TyG-BMI but also found in further ROC analysis that TyG-BMI had the best NAFLD discrimination ability compared with the HbA1c, TG, HDL-C, TyG index, FPG, BMI, WC, TG/HDL-C ratio, TyG-WC and TC. Importantly, the TyG-BMI shows excellent predictive performance in detecting NAFLD in young and middle-aged people (18–30 years: AUC: 0.9258; 95% CI: 0.8908–0.9608; 31–45 years: AUC: 0.9090; 95% CI: 0.9015–0.9164). More importantly, the TyG-BMI is calculated according to the FPG, TG and BMI [13], which can be obtained clinically with simple calculation, is inexpensive and convenient, and conducive to rapid promotion and application in clinical practice.

Compared with previous studies, this study also explored the non-linear correlation, and the results were equally surprising. The results show a stable non-linear relationship between NAFLD and the TyG-BMI before and after adjustment of the generalized additive model. It is exciting that threshold and saturation effects exist in the correlation between NAFLD and the TyG-BMI; a TyG-BMI between 100 and 150 likely indicates a threshold effect point, while a TyG-BMI between 300 and 400 likely suggests that the corresponding NAFLD risk is a saturation effect. As far as I know, this is the first report of the non-linear relationship between NAFLD and the TyG-BMI. The discovery of the threshold effect and saturation effect can provide new ideas for clinical medical workers to prevent and treat NAFLD.

In subgroup analysis, some interesting phenomena were also found in this study. Compared with other age groups, middle-aged people have a higher risk of TyG-BMI-related NAFLD. After further analysis of the baseline information of the study population grouped according to age, middle-aged people were found to lack physical exercise compared with other age groups [14.37% (31–45 years) vs 18.29% (18–30 years), 20.55% (46–60 years), and > 33.47% (60 years); Supplementary Table 3]. Presently, exercise is the only recognized method to prevent and treat NAFLD [8, 31, 32]. A lack of physical exercise can not only easily lead to NAFLD but also easily lead to disorders of blood lipids, blood pressure and blood glucose metabolism and then increase the risk of cardiovascular and cerebrovascular diseases, diabetes and neuropsychiatric diseases [33–35]. The cause of this situation may be related to the current social structure and division of labour. With the further aggravation of ageing and growing economic needs of families, the pressure on middle-aged people is also increasing [36, 37]. In addition to age, a significant interaction was found between the BMI and TyG-BMI, in which the risk of NAFLD associated with TyG-BMI was higher in non-obese people than in overweight and obese people. After further analysis of the baseline information of subjects for BMI stratification (Supplementary Table 4), it can be seen that there were more non-obese women than men in this study. Sex differences in non-obese NAFLD have also been noted in some previous studies [38, 39], likely related to the definition of non-obesity being mainly distinguished by the BMI. Generally, female individuals tend to have more subcutaneous and visceral fat [40, 41], and the BMI alone does not provide a complete picture of this information [39]. Recently, related studies on non-obese NAFLD have found that non-obese people are more prone to metabolic disorders [42, 43]. Combined with the subgroup analysis content of this study, the research team agreed that non-obese NAFLD should be given more attention.

### Study strengths and limitations

Several strengths worth mentioning in this research. 1) This report is the first to study TyG-BMI and the risk of NAFLD in the general population. The population of this study is not only limited to non-obese people but also overweight and obese people. The research data of the general population provide more reference information for follow-up research. 2) This report is also the first to show a non-linear relationship between NAFLD and the TyG-BMI with threshold and saturation effects between them, a finding that will be very helpful help to clinicians. 3) This study included 14,251 subjects, representing a large sample size, and performed using strict statistical adjustment and sensitivity analysis. The results of the data analysis are considered relatively stable.

In addition to research strengths, some research limitations should be highlighted. 1) The design of this study was cross-sectional. Thus, it can only explain the correlation between NAFLD and the TyG-BMI; the causal association between the two must be confirmed by further longitudinal studies. Inevitably, some covariables cannot be measured or evaluated in observational studies; thus, residual confounding may occur [44]. However, this study fully adjusted the variables under the existing conditions, and the main results were similar to previous research results [14–16]. Therefore, the results of this study can be considered to be relatively stable. 2) The diagnosis of NAFLD in this study was conducted according to abdominal ultrasound, while the gold standard for the diagnosis of NAFLD was liver biopsy [1, 2], leading to some patients not having mild hepatic steatosis. However, for more than thousands or even tens of thousands of subjects, liver biopsies would be unethical. 3) Measurement data of IR were lacking in this study, and IR may be the key factor leading to NAFLD with a higher TyG-BMI. Although TyG-BMI has better predictive performance than the TyG index in identifying IR by reviewing the historical literature [13, 27, 28], this conclusion has not been verified in this study; 4) the results of this study are primarily applicable to the assessment of the risk of NAFLD in the general population, but can not further distinguish between nonalcoholic steatohepatitis and NAFL.

## Conclusion

Overall, this study confirmed a statistically significant correlation between the increase in the TyG-BMI and NAFLD risk in the general population. A TyG-BMI between 100 and 150 likely indicates a threshold effect point, while a TyG-BMI between 300 and 400 likely suggests that the corresponding NAFLD risk is saturated. The present study further enhanced the application value of the TyG-BMI in the diagnosis of NAFLD and indicated a simpler and more economical method to prevent and treat NAFLD.

## Supplementary Information

**Additional file 1: Supplementary Table 1.** Collinearity diagnostics steps. **Supplementary Table 2.** Association between TyG-BMI and baseline variables. **Supplementary Table 3.** Baseline characteristics of age groups. **Supplementary Table 4.** Baseline characteristics of BMI groups.

## Data Availability

The datasets that support the conclusions of this article can be found in the Dryad repository.

## Abbreviations

WC: Waist circumference
AUC: Area under the curve
IR: Insulin resistance
GGT: Gamma-glutamyl transferase
CI: Confidence interval
FPG: Fasting blood glucose
S/BBP: Systolic/diastolic blood pressure
BMI: Body mass index
ALT: Alanine aminotransferase
HbA1c: Hemoglobin A1c
AST: Aspartate aminotransferase
TyG index: Triglyceride-glucose index
TC: Total cholesterol
SD: Standard deviation
TG: Triglyceride
OR: Odds ratio
TyG-BMI: Triglyceride glucose-body mass index
NAFLD: Nonalcoholic fatty liver disease
HDL-C: High-density lipoprotein cholesterol
