# Supplementary material for: Usefulness of the triglyceride glucose-body mass index in evaluating nonalcoholic fatty liver disease: insights from a general population
Source: Lipids Health Dis. 2021 Jul 28;20:77. doi: 10.1186/s12944-021-01506-9 (PMC8317400; doi:10.1186/s12944-021-01506-9)
Supplement: Supplementary file 1 — Additional file 1: Supplementary Table 1. Collinearity diagnostics steps. Supplementary Table 2. Association between TyG-BMI and baseline variables. Supplementary Table 3. Baseline characteristics of age groups. Supplementary Table 4. Baseline characteristics of BMI groups. [file 12944_2021_1506_MOESM1_ESM.docx]

Supplementary Table 1: Collinearity diagnostics steps.

| VIF | Step 1 | Step 2 | Step 3 | Step 4 | Step 5 | Step 6 |
| --- | --- | --- | --- | --- | --- | --- |
| Sex | 3.3 | 3.3 | 3.3 | 3.3 | 3.3 | 3.3 |
| Age | 1.4 | 1.4 | 1.4 | 1.4 | 1.3 | 1.3 |
| BMI | 267.1 | NA | NA | NA | NA | NA |
| WC | 5.9 | 5.9 | 5.8 | 5.3 | 5.3 | NA |
| ALT | 4.2 | 4.1 | 4.1 | 4.1 | 4.1 | 4.1 |
| AST | 3.3 | 3.3 | 3.3 | 3.3 | 3.3 | 3.3 |
| Weight | 176.6 | 148.1 | NA | NA | NA | NA |
| Habit of exercise | 1 | 1 | 1 | 1 | 1 | 1 |
| GGT | 1.5 | 1.5 | 1.5 | 1.5 | 1.5 | 1.5 |
| HDL-C | 1.9 | 1.9 | 1.9 | 1.9 | 1.9 | 1.9 |
| TC | 1.6 | 1.6 | 1.6 | 1.5 | 1.5 | 1.5 |
| TG | 6.6 | 5.6 | 5.3 | 2.5 | 2.5 | 2.1 |
| FPG | 1.7 | 1.7 | 1.6 | 1.5 | 1.5 | 1.5 |
| TyG | 61.2 | 27.2 | 8.3 | NA | NA | NA |
| HbA1c | 1.2 | 1.2 | 1.2 | 1.2 | 1.2 | 1.2 |
| Drinking status | 1.2 | 1.2 | 1.2 | 1.2 | 1.2 | 1.2 |
| Smoking status | 1.4 | 1.4 | 1.4 | 1.4 | 1.4 | 1.4 |
| SBP | 5.5 | 5.5 | 5.5 | 5.5 | 1.4 | 1.4 |
| DBP | 5.6 | 5.6 | 5.6 | 5.6 | NA | NA |
| Height | 54.1 | 45.2 | 2.9 | 2.8 | 2.8 | 2.4 |

VIF = 1/(1-R^2^). Abbreviations as in Table ​1.

Supplementary Table 2: Association between TyG-BMI and baseline variables.

|  | Statistics | β (95%CI) | *P*-value |
| --- | --- | --- | --- |
| Sex | 7411 (52.00%) | 28.39 (27.36, 29.43) | <0.0001 |
| Age | 43.53 ± 8.89 | 0.56 (0.50, 0.62) | <0.0001 |
| BMI | 22.06 ± 3.14 | 10.23 (10.16, 10.30) | <0.0001 |
| WC | 76.19 ± 9.10 | 3.20 (3.16, 3.23) | <0.0001 |
| ALT | 19.76 ± 14.47 | 0.98 (0.95, 1.02) | <0.0001 |
| AST | 18.23 ± 8.67 | 0.87 (0.81, 0.93) | <0.0001 |
| Weight | 60.26 ± 11.61 | 2.46 (2.43, 2.48) | <0.0001 |
| Height | 164.80 ± 8.48 | 1.05 (1.00, 1.12) | <0.0001 |
| Habit of exercise | 2470 (17.33%) | -1.59 (-3.09, -0.10) | 0.0368 |
| GGT | 19.13 ± 16.13 | 0.76 (0.73, 0.80) | <0.0001 |
| HDL-C | 1.46 ± 0.40 | -43.66 (-44.88, -42.45) | <0.0001 |
| TC | 5.12 ± 0.87 | 12.06 (11.44, 12.68) | <0.0001 |
| TG | 0.89 ± 0.63 | 36.68 (36.02, 37.34) | <0.0001 |
| FPG | 5.15 ± 0.41 | 36.54 (35.30, 37.77) | <0.0001 |
| TyG | 8.01 ± 0.64 | 40.49 (39.91, 41.07) | <0.0001 |
| HbA1c | 5.18 ± 0.32 | 19.23 (17.49, 20.97) | <0.0001 |
| Drinking status |  |  |  |
| Non or small | 11805 (82.84%) | Ref |  |
| Light | 1758 (12.34%) | 7.67 (5.95, 9.39) | <0.0001 |
| Moderate | 688 (4.83%) | 14.86 (12.23, 17.50) | <0.0001 |
| Smoking status |  |  |  |
| Non | 8746 (61.37%) | Ref |  |
| Former | 2559 (17.96%) | 16.52 (15.05, 17.99) | <0.0001 |
| Current | 2946 (20.67%) | 18.55 (17.16, 19.95) | <0.0001 |
| SBP | 113.93 ± 14.82 | 1.12 (1.08, 1.15) | <0.0001 |
| DBP | 71.12 ± 10.38 | 1.58 (1.53, 1.63) | <0.0001 |

Abbreviations as in Table ​1.

Supplementary Table 3: Baseline characteristics of age groups.

|  | Age groups | | | |  |
| --- | --- | --- | --- | --- | --- |
|  | >60 | 46-60 | 31-45 | 18-30 | *P*-value |
| No. of participants | 487 | 4909 | 8210 | 645 |  |
| Height (cm) | 161.47 (8.32) | 163.30 (8.32) | 165.86 (8.39) | 165.06 (8.62) | <0.001 |
| Weight (kg) | 57.96 (9.37) | 59.88 (10.60) | 60.85 (12.19) | 57.50 (12.32) | <0.001 |
| BMI (kg/m^2^) | 22.15 (2.60) | 22.35 (2.93) | 21.97 (3.25) | 20.95 (3.25) | <0.001 |
| WC cm | 78.32 (8.34) | 77.34 (8.77) | 75.72 (9.20) | 71.66 (8.89) | <0.001 |
| NAFLD | 81 (16.63%) | 993 (20.23%) | 1392 (16.95%) | 41 (6.36%) | <0.001 |
| ALT (IU/L) | 18 (14-22) | 17 (13-22) | 16 (12-23) | 15 (11-20) | <0.001 |
| AST (IU/L) | 20 (17-23) | 18 (15-21) | 17 (14-20) | 16 (13-19) | <0.001 |
| GGT (IU/L) | 17 (13-25) | 15 (12-22) | 14 (11-20.75) | 13 (10-17) | <0.001 |
| HDL-C (mmol/L) | 1.42 (0.42) | 1.46 (0.42) | 1.46 (0.39) | 1.52 (0.38) | <0.001 |
| TC (mmol/L) | 5.46 (0.84) | 5.42 (0.86) | 4.97 (0.82) | 4.58 (0.77) | <0.001 |
| TG (mmol/L) | 0.87 (0.63-1.24) | 0.82 (0.58-1.21) | 0.67 (0.45-1.03) | 0.52 (0.37-0.72) | <0.001 |
| FPG (mmol/L) | 5.29 (0.41) | 5.21 (0.42) | 5.11 (0.40) | 4.98 (0.38) | <0.001 |
| TyG | 8.23 (0.56) | 8.15 (0.59) | 7.94 (0.66) | 7.64 (0.59) | <0.001 |
| TyG-BMI | 182.60 (27.34) | 182.94 (31.59) | 175.57 (36.00) | 160.87 (33.24) | <0.001 |
| HbA1c (%) | 5.40 (5.10-5.60) | 5.20 (5.00-5.50) | 5.10 (4.90-5.40) | 5.10 (4.90-5.20) | <0.001 |
| SBP (mmHg) | 120.99 (14.64) | 116.60 (15.63) | 112.34 (14.03) | 108.63 (13.30) | <0.001 |
| DBP (mmHg) | 75.27 (9.24) | 73.57 (10.69) | 69.82 (9.97) | 65.90 (8.77) | <0.001 |
| Sex |  |  |  |  | <0.001 |
| Female | 172 (35.32%) | 2410 (49.09%) | 3885 (47.32%) | 373 (57.83%) |  |
| Male | 315 (64.68%) | 2499 (50.91%) | 4325 (52.68%) | 272 (42.17%) |  |
| Habit of exercise | 163 (33.47%) | 1009 (20.55%) | 1180 (14.37%) | 118 (18.29%) | <0.001 |
| Drinking status |  |  |  |  | <0.001 |
| Non or small | 359 (73.72%) | 3937 (80.20%) | 6911 (84.18%) | 598 (92.71%) |  |
| Light | 79 (16.22%) | 662 (13.49%) | 977 (11.90%) | 40 (6.20%) |  |
| Moderate | 49 (10.06%) | 310 (6.31%) | 322 (3.92%) | 7 (1.09%) |  |
| Smoking status |  |  |  |  | <0.001 |
| Non | 248 (50.92%) | 2892 (58.91%) | 5167 (62.94%) | 439 (68.06%) |  |
| Former | 168 (34.50%) | 986 (20.09%) | 1323 (16.11%) | 82 (12.71%) |  |
| Current | 71 (14.58%) | 1031 (21.00%) | 1720 (20.95%) | 124 (19.22%) |  |

Abbreviations as in Table ​1.

Supplementary Table 4: Baseline characteristics of BMI groups.

|  | BMI groups | | | |  |
| --- | --- | --- | --- | --- | --- |
|  | ≥28 | ≥24, <28 | ≥18.5, <24 | <18.5 | *P*-value |
| No. of participants | 644 | 2726 | 9336 | 1545 |  |
| Age (years) | 42.75 (8.17) | 44.69 (8.57) | 43.67 (8.94) | 40.97 (8.94) | <0.001 |
| WC (cm) | 95.10 (7.13) | 84.95 (5.18) | 74.24 (6.23) | 64.61 (4.38) | <0.001 |
| NAFLD | 485 (75.31%) | 1172 (42.99%) | 847 (9.07%) | 3 (0.19%) | <0.001 |
| Weight (kg) | 84.41 (10.77) | 71.29 (7.76) | 57.75 (7.78) | 45.90 (4.71) | <0.001 |
| Height (cm) | 167.08 (8.65) | 167.02 (8.37) | 164.50 (8.49) | 161.71 (7.26) | <0.001 |
| ALT (IU/L) | 29.00 (20.00-45.00) | 22.00 (16.00-31.00) | 16.00 (12.00-20.00) | 14.00 (11.00-17.00) | <0.001 |
| AST (IU/L) | 21.00 (17.00-28.00) | 19.00 (15.00-23.00) | 17.00 (14.00-20.00) | 16.00 (14.00-20.00) | <0.001 |
| GGT (IU/L) | 23.00 (16.00-35.00) | 20.00 (14.00-29.00) | 14.00 (11.00-19.00) | 12.00 (10.00-15.00) | <0.001 |
| HDL-C (mmol/L) | 1.17 (0.27) | 1.24 (0.31) | 1.50 (0.39) | 1.72 (0.41) | <0.001 |
| TC (mmol/L) | 5.49 (0.84) | 5.33 (0.88) | 5.09 (0.85) | 4.84 (0.83) | <0.001 |
| TG (mmol/L) | 1.25 (0.89-1.78) | 1.04 (0.71-1.54) | 0.68 (0.46-0.99) | 0.49 (0.36-0.69) | <0.001 |
| FPG (mmol/L) | 5.42 (0.36) | 5.33 (0.37) | 5.11 (0.40) | 4.93 (0.39) | <0.001 |
| TyG | 8.59 (0.53) | 8.39 (0.59) | 7.93 (0.60) | 7.56 (0.51) | <0.001 |
| TyG-BMI | 259.12 (24.36) | 213.98 (18.37) | 168.94 (20.08) | 132.52 (10.91) | <0.001 |
| HbA1c (%) | 5.34 (0.34) | 5.24 (0.33) | 5.16 (0.31) | 5.14 (0.30) | <0.001 |
| SBP (mmHg) | 130.29 (14.41) | 121.70 (14.24) | 112.10 (13.48) | 104.52 (12.59) | <0.001 |
| DBP (mmHg) | 81.87 (9.94) | 76.61 (9.96) | 69.82 (9.61) | 64.81 (8.33) | <0.001 |
| Sex |  |  |  |  | <0.001 |
| Female | 197 (30.59%) | 713 (26.16%) | 4709 (50.44%) | 1221 (79.03%) |  |
| Male | 447 (69.41%) | 2013 (73.84%) | 4627 (49.56%) | 324 (20.97%) |  |
| Habit of exercise | 82 (12.73%) | 438 (16.07%) | 1727 (18.50%) | 223 (14.43%) | <0.001 |
| Drinking status |  |  |  |  | <0.001 |
| Non or small | 538 (83.54%) | 2159 (79.20%) | 7706 (82.54%) | 1402 (90.74%) |  |
| Light | 81 (12.58%) | 366 (13.43%) | 1188 (12.72%) | 123 (7.96%) |  |
| Moderate | 25 (3.88%) | 201 (7.37%) | 442 (4.73%) | 20 (1.29%) |  |
| Smoking status |  |  |  |  | <0.001 |
| Non | 344 (53.42%) | 1321 (48.46%) | 5861 (62.78%) | 1220 (78.96%) |  |
| Former | 122 (18.94%) | 673 (24.69%) | 1640 (17.57%) | 124 (8.03%) |  |
| Current | 178 (27.64%) | 732 (26.85%) | 1835 (19.66%) | 201 (13.01%) |  |

Abbreviations as in Table ​1.
